# Are probiotics beneficial for obese patients with major depressive disorder? Opinion for future implications and strategies

**DOI:** 10.3389/fnut.2023.1205434

**Published:** 2023-05-31

**Authors:** Theresia M. D. Kaunang, Aurielle Annalicia Setiawan, Nelly Mayulu, Ivena Leonita, Afredo Wijaya, Vincentius Mario Yusuf, Msy Firyal Nadya Al Mahira, Dewangga Yudisthira, William Ben Gunawan, Nurpudji Astuti Taslim, Athaya Febriantyo Purnomo, Nindy Sabrina, Nurlinah Amalia, Happy Kurnia Permatasari, Fahrul Nurkolis

**Affiliations:** ^1^Department of Mental Health Sciences, Faculty of Medicine, Sam Ratulangi University-Prof. R. D. Kandou General Hospital, Manado, Indonesia; ^2^Medical Study Programme, Faculty of Medicine, Brawijaya University, Malang, Indonesia; ^3^Department of Nutrition, Universitas Muhammadiyah Manado, Manado, Indonesia; ^4^Alumnus of Nutrition Science, Faculty of Medicine, Diponegoro University, Semarang, Indonesia; ^5^Division of Clinical Nutrition, Department of Nutrition, Faculty of Medicine, Hasanuddin University, Makassar, Indonesia; ^6^Department of Urology, Faculty of Medicine, Universitas Brawijaya - Saiful Anwar General Hospital, Malang, Indonesia; ^7^Department of Oncology, University of Oxford, Oxford, United Kingdom; ^8^Nutrition Program, Faculty of Food Technology and Health, Sahid University of Jakarta, South Jakarta, Indonesia; ^9^Biomedical Science Master Program, Faculty of Medicine, Brawijaya University, Malang, Indonesia; ^10^Department of Biochemistry and Biomolecular, Faculty of Medicine, Brawijaya University, Malang, Indonesia; ^11^Department of Biological Sciences, State Islamic University of Sunan Kalijaga (UIN Sunan Kalijaga), Yogyakarta, Indonesia

**Keywords:** obesity, probiotics, synbiotics, major depressive disorder, psychobiotics, gut microbiota

## 1. Introduction

Mental health has become one of the main topics in discussing the burden of disease in recent decades. This issue is a major cause of high morbidity rates, an important risk factor for premature death, decreased daily productivity, and diseases with high costs (1, 2). The Global Burden of Diseases, Injuries, and Risk Factors Study (GBD) in 2019 stated that mental health problems that commonly occur are depression and anxiety disorders. Both ranked among the top 25 as major diseases worldwide (3). It was estimated that 418 million disability-adjusted life years and an economic burden of $5 trillion might be attributed to mental diseases, with a more than 3-fold increase over previous projections (1).

Major Depressive Disorder (MDD) is a mood disorder characterized by feelings of depression (sadness, irritability, emptiness) or loss of pleasure or interest in activities (4). Approximately 280 million people worldwide, or an estimated 5% of adults, suffer from MDD (3). There are several lifestyle risk factors that can cause someone to develop MDD, including physical activity, healthy diet, sleep duration, smoking habit, and screen time (5), with obesity being one of the main causes. Obesity itself is also a disease with a high incidence, affecting 600 million people worldwide, and at least 2.6 million people die from obesity-related causes each year (4). A prospective study found a 30% increased incidence of MDD in individuals with obesity (6). From a psychosocial perspective, obesity leads to self-devaluation, particularly in environments with high social expectations and beauty standards. From a biomolecular perspective, both MDD and obesity are disorders associated with dysregulation of the stress system, involving changes in the hypothalamic-pituitary-adrenal (HPA) axis, inflammation, oxidative stress, and endocrine dysfunction (7). Depression in studies is measured using various types of scales, such as BDI (Beck Depression Index), Hamilton Rating Scale for Depression (HAMD/HDRS), Montgomery-Åsberg Depression Rating Scale (MADRS), etc., (8).

Currently, several treatment options—Such as behavioral weight management and cognitive behavioral therapy—are available for obesity and depression (9). However, evidence-based management that simultaneously targets both conditions has not been thoroughly investigated. The main therapy usually provided is subjective lifestyle education that cannot be quantitatively assessed. Supplementation therapy with zinc and vitamin D has been found to improve Beck Depression Inventory (BDI-II) scores in patients with obesity and MDD; however, this intervention does not lead to weight loss (7, 10). On the other hand, long-term antidepressant therapy (>25 months) increases the risk of type 2 diabetes (11). Some antidepressant classes, such as monoamine oxidase inhibitors (MAOIs), can cause weight gain, and tricyclic antidepressants (TCAs) can cause hyperglycemia, both of which are counterproductive and dangerous in patients with diabetes (10, 11).

Recent studies have found that probiotics can be used as a treatment for mental health patients with metabolic problems (12). Probiotics with specific functions for mental health are known as psychobiotics (13). Some studies have found that probiotics can reduce symptoms of depression and improve the condition of patients (14). Gut probiotics can produce vitamins, such as vitamin B (Folic Acid and Pyridoxine), which, when deficient, may be involved in the etiology of depression and hinder the treatment of depression in patients (15, 16). In addition, pyridoxine can also reduce symptoms of depression and anxiety (17). Probiotics also contain Short Chain Fatty Acids (SCFA) as probiotic metabolites that play a role in anti-inflammatory processes that lead to increased production of IL-8 (18, 19). On the other hand, SCFA functions as anti-obesity by increasing insulin sensitivity, fatty acid oxidation, and reducing fat accumulation (20). Probiotics can also improve the general condition of patients and have minimal side effects, thus improving the treatment satisfaction of patients with depression, which has been a concern (14). A study conducted by Gawlik-Kotelnicka in 2021 clearly stated that psychobiotics can be an adjunctive therapy for MDD (12). Furthermore, another study suggests that probiotics have a clear positive effect on individuals with obesity (21). This is something interesting to further discuss and synthesize the exact relationship between probiotic supplementation and obesity and MDD.

At present, clinical research on the use of probiotics in obese patients with MDD is still contradicting but remain a hot topic in the utilization of probiotics. Therefore, this opinion article aims to provide critical opinions and summarize evidence-based clinical research on the potential of probiotics as a supplementary treatment for depression in obese patients.

## 2. Health beneficial effects of probiotics

Probiotics are living microorganisms that, when administered in sufficient amounts, provide benefits to the health of the host (15). Probiotics consist of many types of microbes. The most commonly used microbes as probiotics are species from the genera Lactobacillus, Bifidobacterium, and Saccharomyces (16). Probiotics must be administered in a live state and in an effective dosage (15).

There are many potential sources to obtain probiotics and each microorganism has different sources. Lactobacillus can be isolated from fruits and fermented foods (17). Artisanal soft cheese can also be a source of isolation for Lactobacillus, Lactococcus, Leuconostoc, and Enterococcus genera (18). *L. paracase*i can be found in human breast milk (19). In addition, other potential sources of isolation include spoiled fruits such as grapes, apples, strawberries, tomatoes, cucumbers, and traditional fermented foods from Korea such as Kimchi and Jangajji (22, 23).

Probiotics have wide range of benefits due to the broad range of probiotic activities that can produce physiological benefits. However, probiotics are not a single substance, and as a biological entity, probiotics have the potential to work in varied ways. Some mechanisms of probiotic action that have been discovered include modulation of the immune system, interaction with the microbiota of the digestive system, production of organic acids, production of small molecules with systemic effects, and production of enzymes (15, 24). Research has also found that probiotics have anti-pathogen activities, which are considered the most beneficial effects (25). This is because unlike antibiotics, probiotics do not alter the gut microbiota population. Probiotics are also found to have anti-diabetic activity, as diabetes is believed to occur due to an imbalance of gut microbiota. In addition, probiotics have been found to have anti-inflammatory, anti-allergic, angiogenic, and even anti-cancer effects (26). However, there is still much unknown about the mechanisms of probiotic action and further research is needed.

Probiotics have been studied for their use in various conditions, including acute diarrhea, constipation, prevention of upper respiratory and gastrointestinal tract infections, ulcerative colitis, management of lactose intolerance symptoms, and obesity (15). Nowadays, the diet of the population is often high in fat, preservatives, carbohydrates, and low in fiber. This causes disturbances in the composition of the intestinal microbiota and leads to inflammation and the emergence of metabolic disorders such as obesity and diabetes. Therefore, probiotics are believed to be able to address the disturbances in the composition of the intestinal microbiota in these cases (27). However, the benefits of probiotics are still debated. Some recent studies have shown that the use of probiotics can reduce body weight, BMI, waist circumference, and metabolic parameters in systematic reviews conducted by Boricha et al. (28). A systematic review by da Silva Pontes et al. found that the use of probiotics in overweight and obese patients can reduce body adiposity and cardiovascular risk markers, including inflammatory markers, insulin levels, total cholesterol, and LDL (29). Meta-analysis conducted by Wang et al. also found similar results, with improvement in lipid profiles with the use of probiotics in overweight or obese patients (30). Probiotics have promising potential as part of the therapy for obese patients.

## 3. The role of probiotics in treating MDD in obese patients

MDD is a multifactorial disease, with factors such as stress, genetics, and psychosocial factors playing a role. Symptoms of MDD include depressive affect, loss of interest, changes in weight or appetite, changes in sleep patterns (insomnia or hypersomnia), psychomotor agitation or retardation, decreased energy, feelings of worthlessness or uselessness, difficulty concentrating, and suicidal ideation or suicide attempt (31). The pathophysiology of this disease is still unclear, but it is believed that abnormalities in neurotransmitter activity, particularly serotonin (5-HT) in the central nervous system, play a role (32).

Interestingly, MDD is often found to be comorbid with obesity (33). The pathophysiology of MDD can be linked to changes in gut microbiota. This is because gut microbiota interacts with the brain through neuroendocrine, neuroimmune, and neural pathways (34). Obesity is also associated with dysregulated gut microbiota composition, usually induced by a high-fat diet (HFD) (35). HFD is known to cause chronic systemic inflammation in animals and humans, where increased expression of IL-1b, IL-6, and TNF-a is found, and reduced expression of tight junction proteins in the choroid plexus and blood-brain barrier (BBB) in mice, disrupting BBB integrity and affecting brain function. Increased BDNF and decreased tyrosine hydroxylase levels, limiting dopamine synthesis, were also found in mice fed with HFD (36).

### 3.1. Obesity and MDD: pathophysiology and relationship in relation to gut microbiome

The gut microbiota ecosystem consists of thousands of bacterial strains that support metabolic homeostasis and various physiological responses such as energy requirements, vitamin synthesis, food absorption and digestion, stress, and food consumption patterns (37). Several studies have shown that obese patients experience an increased ratio of Firmicutes/Bacteroidetes accompanied by decreased microbial diversity compared to physiological conditions (38). Firmicutes and Bacteroidetes are dominant bacterial phyla that contribute to 90% of the gut microbiota population. Alterations in gut microbiota composition often triggered by a high-fat diet contribute to low-grade chronic inflammation that occurs in obesity (39). This inflammation is characterized by increased adipokine production by white adipose tissue accompanied by decreased inhibitory Treg lymphocytes, infiltration of immune cells such as macrophages, which further activate pro-inflammatory cytokines, leading to insulin resistance (40).

Gut dysbiosis is also found to be relevant to mood changes due to bidirectional interactions with the HPA-Axis. The immune and inflammatory status of the gut microbiota can affect the modulation of neuroactive components such as dopamine, glutamate, GABA, and serotonin (41). In situations of chronic stress, microglia become hyperactive and produce a series of inflammatory pathways through cytokines, MHC 1 and II, and neurotoxic molecules (ROS) (42). Systemic low-grade inflammation has a direct effect on neurogenesis, where pro-inflammatory mediators reduce serotonin and melatonin synthesis while increasing tryptophan catabolites through the activity of indoleamine 2,3-dioxygenase (IDO), resulting in neuronal damage and symptoms closely related to MDD such as malaise and anhedonia (43, 44).

Under conditions of stress, excessive activation of the HPA axis results in uncontrolled cortisol levels, leading to increased gut permeability, also known as leaky gut (45). Imbalance of gut microbiota further leads to decreased intestinal barrier proteins occludin and zonulin-1, and increased gut permeability, where this gut barrier dysfunction facilitates the translocation of gut gram-negative bacteria or lipopolysaccharide (LPS) it produces (46). LPS can bind to TLR-4, resulting in increased pro-inflammatory cytokines and chemokines, leading to systemic inflammation and metabolic endotoxemia (47). This metabolic endotoxemia further results in a 35% decrease in insulin sensitivity, triggering increased calorie intake by patients with high-carbohydrate or high-fat diets (48). LPS also translocates from the gut to the brain and disrupts the blood-brain barrier through depression of tight junctions and anchoring junction proteins in the hippocampus, striatum, and frontal cortex (41).

The composition of gut microbiota communities in obesity (Firmicutes, Proteobacteria, and Tenericutes) has been found to decrease fatty acid oxidation in the liver through inhibition of adenosine monophosphate kinase (AMPK) enzyme, resulting in the accumulation of fat and synthesis of cholesterol and triglycerides (47, 49). Angiopoietin-like protein 4 (ANGPTL4), an adipose tissue molecule that plays a role in inhibiting lipoprotein lipase activity, is also blocked by this group of microbiotas, resulting in increased energy storage in the form of fat (50).

### 3.2. Gut brain-axis and microbiome modulation via probiotic supplementation

**Figure 1:**
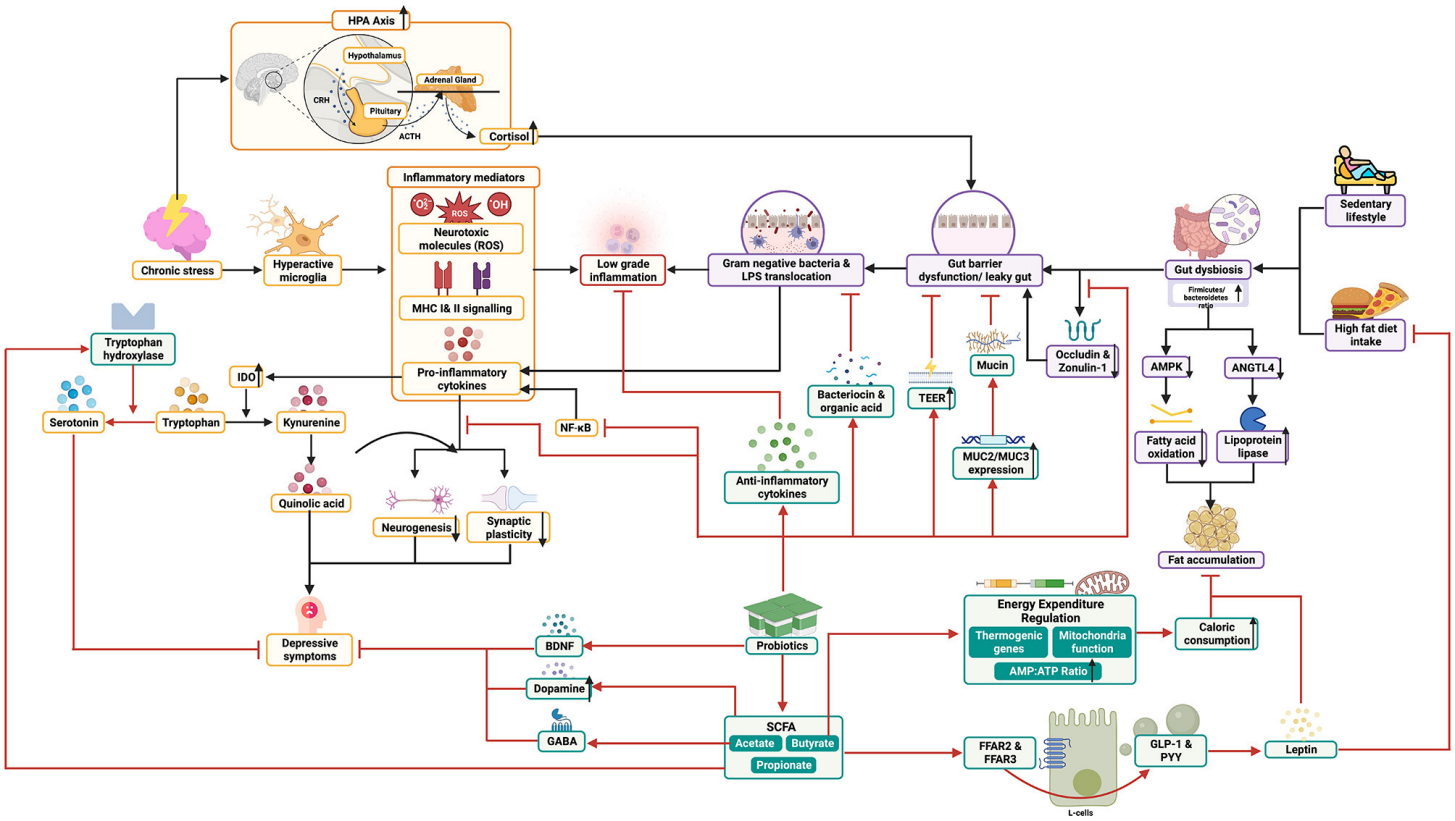
Possible biomechanism of probiotics to treat obese patients with major depressive disorder and their mechanistic pathway.

Approximately 57% of gut microbiota composition is estimated to be influenced by diet, while only 12% is influenced by genetic variation (50). Various studies have shown that intervention with probiotic consumption plays a role in preventing and improving dysbiosis, through the improvement and enhancement of cytoskeleton structure, increased mucin secretion through the activation of secretory proteins MUC2 and MUC3, and phosphorylation of tight junction proteins in the gut barrier (Figure 1) (51, 52). Improvement of dysbiosis is achieved through increased variability of gut microbiota and production of bacteriocin and organic acids, creating an unfavorable environment for the colonization of pathogenic bacteria and their metabolites (53, 54). In addition, probiotic supplementation can increase tightness and restore damaged epithelial barrier function through the improvement of transepithelial electrical resistance (TEER) (55). TEER is a measure of the electrical resistance of epithelium and is used as one of the parameters for epithelial barrier integrity (56).

Immunomodulatory abilities have also been found in probiotics, where increased production of anti-inflammatory cytokines such as IL-10 from dendritic cells and decreased activation of Th-1 cells have been observed (Figure 1) (47). Fermentation products of fiber by probiotics, namely short-chain fatty acids (SCFA) including acetate, propionate, and butyrate, are also believed to play a role in reducing inflammation through inhibition of NF-kB (57). SCFA binding to FFAR2 and FFR3 receptors triggers GLP-1 secretion from intestinal cells, which increases the formation of leptin hormone that reduces appetite, fatty acid oxidation in liver and muscle tissues, reduces cholesterol levels, and improves insulin sensitivity, which is often impaired in obese patients (Figure 1) (46, 58). In addition, probiotic supplementation has been found to reduce excessive HPA axis activation and corticosterone levels, and even normalize BDNF and serotonin levels in animal studies, which has protective effects against depression through increased differentiation, defense, and synaptic plasticity (59, 60).

SCFA has been proven to reduce bile duct pH, which modifies the gut microbiota composition in such a way as to promote the growth of butyrate-producing bacteria (58). Not only that, SCFA also stimulates the secretion of gut hormones such as glucagon-like peptide-1 (GLP-1) and peptide YY (PYY), which aid in signaling satiety sensation, reducing fat deposition, and increasing fat breakdown in the body (Figure 1) (57). SCFA regulates thermogenic genes, mitochondrial function, and AMP to ATP ratio, thus triggering oxidative metabolism in various tissues, which increases calorie consumption (61). Some probiotic strains have also been found to influence genes related to fatty acid metabolism, such as AMPK phosphorylation and increased ANGPTL4, which help reduce fat accumulation in obese patients (Figure 1) (21).

In addition to functioning in the enteroendocrine pathway, SCFA also has neuroactive properties, such as acetate that can cross the blood-brain barrier and enhance GABA neurotransmission, which may help ameliorate neurotransmitter imbalances in major depressive disorder (MDD) (62, 63). On the other hand, butyrate inhibits the activity of orexigenic neurons in the brainstem, and both have protective effects against excessive energy intake and high-fat diet (58, 64). Moreover, SCFA can also increase the expression of tryptophan 5-hydroxylase 1, a key enzyme in serotonin synthesis, and increase tyrosine hydroxylase, an enzyme involved in the biosynthesis of dopamine, adrenaline, and noradrenaline (63).

### 3.3. Clinical evidences

The clinical evidence of the benefits of probiotics on obese patients with MDD is summarized in Tables 1, 2. The studies found that probiotic administration resulted in significant reduction in body weight, depression scores, body fat mass, and waist circumference. In the study by Othmann, significant reductions in depression scores, body weight, and waist circumference were found in the intervention arm (probiotic) and control arm (diet) at the 1-month follow-up, but significant reduction in body fat mass only occurred in the intervention arm (65). In the study by Sanchez, a decrease in BDI scores was found in the intervention arm between phase 1 and 2, and between phase 2 and baseline, whereas no significant changes were observed in the control arm (placebo). As for obesity parameters, there was a greater reduction in body weight in the intervention arm compared to placebo, although there were no significant reductions in both arms (66). Similarly, in the study by Akkasheh, significant reduction in BDI scores was observed in the probiotic group compared to placebo, although changes in obesity parameters were not significant as this study was conducted earlier and involved participants with normal and overweight BMI (67).

**Table 1:** Characteristics of studies and participants.

**No**	**Study**	**Type of study**	**Country**	**Follow-up duration**	**Intervention Group**	**Subject (*n*)**	**Participant characteristics**			
							**Age (years)**	**Gender**	**BMI (kg/m** ^2^ **)**	**Weight (kg)**
1	Othman et al. (65)	Clinical trial	Tunisia	1 month	Probiotic: 1 tablet containing *Bifidobacterium longum, Lactobacillus helveticus, Lactococcus lactis, Streptococcus thermophilus*/day	15	48.73 ± 7.7 (mean ± SD)	Female 93.3% (*n* = 42) Male 6.7% (*n* = 3)	≥30 (no information for mean and SD)	106.09 (no information for SD)
					Diet only (low-carbohydrate and reduced energy diet)	15				103.7 (no information for SD)
2	Sanchez et al. (66)	RCT	Canada	Phase 1 = 12 weeks Phase 2 = 12 weeks	Probiotic: 2 capsules containing LPR (*Lactobacillus rhamnosus*)/day	62	35 ± 10.0 (mean ± SE)	Male:24 Female:38	33.8 ± 3.3 (mean ± SE)	95.1 ± 13.9 (mean ± SE)
					Placebo	63	37 ± 10.0 (mean ± SE)	Male:24 Female:39	33.3 ± 3.2 (mean ± SE)	94.0± 14.9 (mean ± SE)
3	Akkasheh et al. (67)	RCT	Iran	8 weeks	Probiotic: 1 capsule containing *Lactobacillus acidophilus, Lactobacillus casei, Bifidobacterium bifidum*/day	20	38.3 ± 12.1 (mean ± SD)	Female 85% (*n* = 17) Male 15% (*n* = 3)	27.6 ± 6.0 (mean ± SD)	72.6 ± 11.3 (mean ± SD)
					Placebo (starch)	20	36.2 ± 8.2 (mean ± SD)	Female 85% (*n* = 17) Male 15% (*n* = 3)	26.3 ± 4.1 (mean ± SD)	68.0 ± 11.5 (mean ± SD)

BMI, Body Mass Index; RCT, Randomized Controlled Trial.

**Table 2:** Results of studies included for synthesis opinion and summarize evidence-based clinical research on the potential of probiotics as a supplementary treatment for depression in obese patients.

**No**	**Study**	**Intervention Group**	**Subject (*n*)**	**Parameter**		**Results**		**Conclusion**
				**Depression**	**Obesity**	**Depression**	**Obesity**	
1	Othman et al. (65)	Probiotic: 1 tablet containing *Bifidobacterium longum, Lactobacillus helveticus, Lactococcus lactis, Streptococcus thermophilus*/day	15	HAD	Weight (kg) Fat mass (kg) Lean mass (kg) Waist circumference (cm)	HAD T0 = 11.5 T1= 8.9 (*p =* 0.001)	Weight (kg) T0 = 106.09 T1 = 104.4 (*p =* 0.02) Fat mass (kg) T0 = 47.5 T1 = 45.01 (*p =* 0.001) Lean mass (kg) T0=55.5 T1=56.4 (*p =* 0.08) Waist circumference (cm) T0 = 122 T1 = 119 (*p =* 0.001)	Significant decrease in HAD scores, weight, fat mass, and waist circumference
		Diet only (low-carbohydrate and reduced energy diet)	15			HAD T0 = 12.4 T1 = 9.9 (*p =* 0.001)	Weight (kg) T0 = 103.7 T1 = 101.2 (*p =* 0.001) Fat mass (kg) T0 = 46.7 T1 = 44.8 (*p =* 0.07) Lean mass (kg) T0 = 54.06 T1 = 53.5 (*p =* 0.3) Waist circumference (cm) T0 = 119 T1 = 117.3 (*p =* 0.01)	Significant decrease in HAD scores, weight, and waist circumference
2	Sanchez et al. (66)	Probiotic: 2 capsules containing LPR (*Lactobacillus rhamnosus*)/day	62	BDI	Weight (kg)	ΔBDI (mean ± SE) Phase 1-baseline: 0.1 ± 2.9 ΔBDI Phase 2-Phase 1: −1.3 ± 2.7 (*p* < 0.05) ΔBDI Phase 2-baseline: −1.5 ± 3.0 (*p* < 0.05)	ΔBDI (mean ± SE) Phase 1-baseline: −4.2± 3.2 ΔPhase 2-baseline: −5.3 ± 4.3	Significant decrease in BDI scores in phase 2-phase 1 and phase 2-baseline
		Placebo	63			ΔBDI (mean ± SE) Phase 1-baseline: −0.6 ± 3.1 ΔBDI Phase 2-Phase 1: 0.6 ± 3.2 ΔBDI Phase 2-baseline: 0.5 ± 3.3	ΔBDI (mean ± SE) Phase 1-baseline: −3.4± 2.9 ΔBDI Phase 2-baseline: −3.9 ± 4.2	
3	Akkasheh et al. (67)	Probiotic: 1 capsule containing *Lactobacillus acidophilus, Lactobacillus casei, Bifidobacterium bifidum*/day	20	BDI	Weight (kg)	ΔBDI (mean ± SD) -5.7 ± 6.4 (*p =* 0.001)	Weight (mean ± SD) T0 = 72.6 ± 11.3 (*p* = 0.21) T1 = 72.5 ± 11.1 (*p* = 0.28) ΔT = −0.1 ± 1.6 (*p* = 0.26)	Significant decrease in BDI score compared with placebo (*p =* 0.001). Baseline and end-of-trial means of weight and BMI were not significantly different between probiotic supplements and placebo groups. BMI includes normal, overweight, and obese individuals
		Placebo (starch)	20			ΔBDI (mean ± SD) −1.5 ± 4.8 (*p =* 0.001)	Weight (mean ± SD) T0 = 68.0 ± 11.5 (*p* = 0.21) T1 = 68.7 ± 10.5 (*p* = 0.28) ΔT = 0.7 ± 2.7 (*p* = 0.26)	

BDI, Beck Depression Index; BMI, Body Mass Index; HAD, Hospital Anxiety and Depression Scale; RCT, Randomized Controlled Trial; T0, Baseline; T1, Follow up.

In other studies that are not included in this table, such as the study by Kazemi, although participants with normal and overweight status were included, significant reduction in BDI scores was found in the probiotic intervention group compared to placebo, while the reduction in BDI scores for the prebiotic group was not significant compared to placebo or probiotic (68). The efficacy of probiotics in managing depression has also been investigated in a systematic review by Wallace, which showed that daily consumption of probiotic supplements has a positive effect in improving mood, cognitive symptoms, and anxiety symptoms in MDD without serious side effects (14). Meanwhile, the positive effects of probiotics in improving obesity status were reported in a study by Wiciński, which found improvements in anthropometric and metabolic parameters (21). A meta-analysis study by López-Moreno also suggested a favorable impact of probiotics in modulating gut microbiota dysbiosis for improvement in obesity status (69).

With the significant reduction in obesity and depression parameters in the probiotic intervention group, the evidence supporting the relationship between probiotic supplementation and improvement in obesity status, which may also improve gut microbiota and subsequently through the gut-brain axis, improve depression status, becomes stronger. Therefore, more clinical studies with standardized research methods, especially in parameters used to measure efficacy, and doses, are needed to further investigate the relationship among these three variables.

## 4. Conclusion and future direction of using probiotics

Previous studies have highlighted the potential use of probiotics for managing depression in obese patients. However, the number of studies conducted in this field is still very limited. Markers for assessing the reduction in disease incidence in previous studies also differ between studies, making it difficult to observe the significant effects of probiotics. Although probiotic supplementation has been shown to be significantly beneficial in improving obesity (not yet assessed in patients with MDD) as reported by the previous systematic review and meta-analysis, the effectiveness and efficiency of probiotics need to be further investigated in terms of dosage and duration of use, so that the expected efficacy can be achieved (70).

Through this opinion article, further clinical research is needed to explore the relationship between probiotics and depression in obese patients, given the increasing potential and trend of probiotics in recent years. In the development of probiotic use, it is also expected that future research will address the possible side effects and toxicity that may occur, resulting in the formulation of probiotics suitable for MDD therapy in obese patients.

## Author contributions

TK, FN, AS, NM, and NT contributed to the conceptualization with the design of the critical opinion study, drafted the manuscript, edited-revised it, and approved the final version of the submitted manuscript. FN, WG, and VY contributed to the software and visualization. NM, FN, NT, WG, HP, NS, and AP contributed to the formal analysis, validation, and supervision. WG, IL, AS, VY, MM, AW, DY, AP, NS, NA, and HP contributed to the writing—original draft preparation, review, and editing. All authors and contributors contributed to the opinion article and approved the submitted version.
